# Massive Temporal Lobe Cholesteatoma

**DOI:** 10.1155/2015/121028

**Published:** 2015-03-04

**Authors:** Pasan Waidyasekara, Samuel A. Dowthwaite, Ellison Stephenson, Sandeep Bhuta, Brent McMonagle

**Affiliations:** ^1^Department of Otolaryngology-Head and Neck Surgery, Gold Coast University Hospital, 1 Hospital Boulevard, Southport, QLD 4215, Australia; ^2^Department of Neurosurgery, Gold Coast University Hospital, 1 Hospital Boulevard, Southport, QLD 4215, Australia; ^3^Department of Medical Imaging, Gold Coast University Hospital, 1 Hospital Boulevard, Southport, QLD 4215, Australia

## Abstract

*Introduction*. Intracranial extension of cholesteatoma is rare. This may occur de novo or recur some time later either contiguous with or separate to the site of the original cholesteatoma. *Presentation of Case*. A 63-year-old female presented to a tertiary referral hospital with a fluctuating level of consciousness, fever, headache, and right-sided otorrhoea, progressing over several days. Her past medical history included surgery for right ear cholesteatoma and drainage of intracranial abscess 23 years priorly. There had been no relevant symptoms in the interim until 6 weeks prior to this presentation. Imaging demonstrated a large right temporal lobe mass contiguous with the middle ear and mastoid cavity with features consistent with cholesteatoma. The patient underwent a combined transmastoid/middle fossa approach for removal of the cholesteatoma and repair of the tegmen dehiscence. The patient made an uneventful recovery and remains well over 12 months later. *Conclusion*. This case presentation details a large intracranial cholesteatoma which had extended through a tegmen tympani dehiscence from recurrent right ear cholesteatoma treated by modified radical mastoidectomy over two decades priorly. There was a completely asymptomatic progression of disease until several weeks prior to this presentation.

## 1. Introduction

Cholesteatomas are epidermal inclusion cysts composed of desquamated debris lined by a keratinized squamous epithelium. They may be congenital or acquired. Congenital cholesteatomas are believed to result from abnormal development of the first branchial groove depositing ectopic ectodermal tissue within the temporal bone or intracranially mainly at the cerebellopontine angle. Acquired cholesteatomas originate at the middle ear or mastoid and are associated with otitis media and eustachian tube dysfunction. The pathogenesis is proposed by four main theories: invagination, basal cell hyperplasia, epithelial invasion, and squamous metaplasia. The invagination theory explains the formation of attic cholesteatomas. Retraction of pars flaccida due to negative pressure of the middle ear results in altered epithelial migration and accumulation of desquamated keratin debris [1]. According to the basal cell hyperplasia theory, an alteration in the basement membrane of the pars flaccida results in inverted epithelial proliferation towards the middle ear leading to the initial formation of an “epithelial cone” [2]. The epithelial invasion theory describes cholesteatoma formation from the migration of tympanic membrane epithelium into the middle ear cleft through a perforation [3]. The squamous metaplasia theory suggests that the simple squamous epithelium of the middle ear cleft transforms to keratinised epithelium [4]. The enlarging mass can perforate the tympanic membrane to give the classic appearance of a cholesteatoma. Cholesteatomas tend to propagate medially and are subject to harbouring microbes, such as* Pseudomonas aeruginosa*,* Staphylococcus aureus*, and* Bacteriodes *spp. [5, 6]. Intracranial extension and infection are rare complications of cholesteatoma.

## 2. Case Report

A 63-year-old female was transferred to a tertiary hospital with fever, frontal headache, right-sided otorrhoea, and brief episodes of impaired consciousness. She had an extensive history of otomastoid disease having previously undergone a right modified radical mastoidectomy and craniotomy 23 years prior to surgical treatment of an infected epitympanic cholesteatoma with intracranial abscess.

Six weeks priorly, she was admitted to a regional hospital with similar symptoms, where she was diagnosed with acute otitis media and probable meningitis. She was treated with intravenous ceftazidime and vancomycin empirically for 3 weeks. A computer tomography (CT) scan and magnetic resonance imaging (MRI) of brain with contrast was performed at this time and showed a right-sided intracranial mass within the temporal lobe. Serial CT scans revealed no progression throughout the admission. The possibility of an abscess or a hydatid cyst had been raised by the radiologist. For reasons which remain unclear, no referral for surgical opinion was requested at that time. She was discharged on a 2-week course of intravenous vancomycin and ciprofloxacin-hydrocortisone ear drops. She presented 16 days later with progressive left sided weakness, worsening frontal headache, and fever. At this time she was transferred to our tertiary centre expediently.

On admission to the tertiary hospital, she was drowsy (GCS 14) with a temperature of 39.2°C. Her facial nerve function was normal, but mild weakness of her left hand was evident. Otoscopy of the right mastoid cavity demonstrated a significant amount of debris and clear fluid draining from the roof of the mastoid cavity, suspicious for cerebrospinal fluid (CSF). Microbial culture was positive for methicillin-resistant* Staphylococcus aureus *(MRSA) and she was commenced on intravenous ceftazidime, metronidazole, and vancomycin.

**Figure 1:**
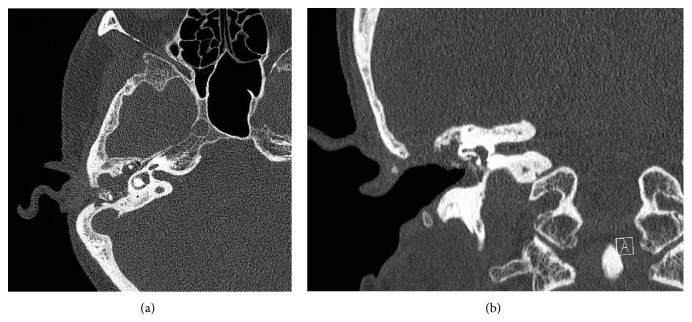
(a) High resolution CT axial image at the level of the lateral semicircular canal demonstrates a fluid filled mastoid cavity, partial erosion of incus, and an intact anterior epitympanic air cell partitioned by the cog. (b) High resolution coronal CT of right temporal bone demonstrating an approximate 9 mm defect involving the superiomedial external ear canal and tegmen mastoideum.

**Figure 2:**
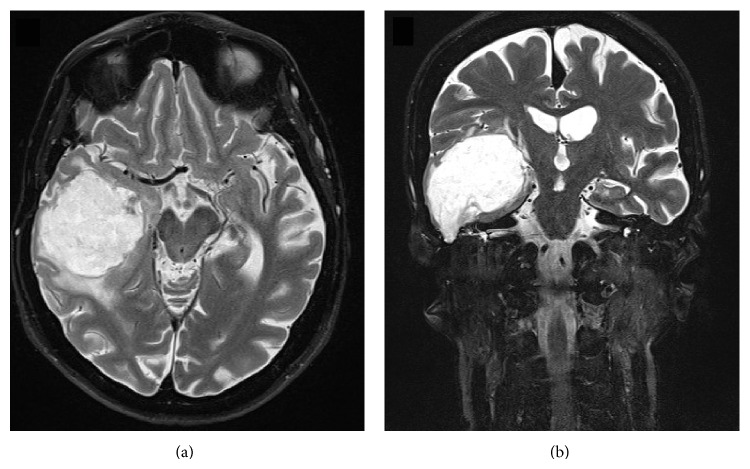
(a) T2-weighted MRI axial image demonstrates a smooth bordered heterogenous hyperintense mass approximately 55 mm × 48 mm with surrounding cytotoxic oedema of brain tissue. (b) T2-weighted MRI coronal image demonstrates the mass extending approximately 52 mm superiorly into the temporal lobe from the right middle ear via tegmen mastoideum defect.

High-resolution CT of the temporal bones demonstrated opacification of the right mastoid cavity with ossicular chain erosion (Figure 1(a)). A 9 mm bony defect of the tegmen tympani was evident (Figure 1(b)). T2-weighted axial MRI scans showed a well demarcated heterogeneous mass approximately 55 mm × 48 mm centred within the right temporal lobe extending from the right tegmen defect with cytotoxic oedema of the surrounding brain tissue (Figures 2(a) and 2(b)).

**Figure 3:**
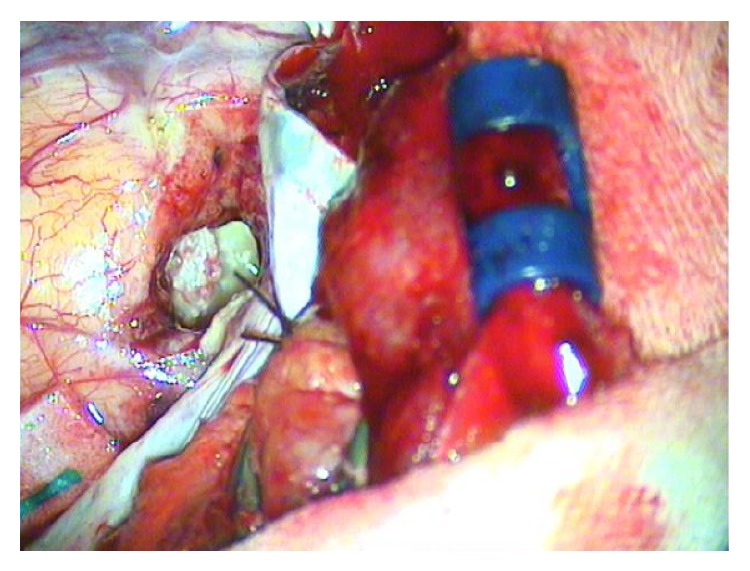
Temporal craniectomy and corticotomy demonstrates a pearl white mass lesion consistent with cholesteatoma in the middle cranial fossa.

A combined transmastoid/middle cranial fossa approach was undertaken under general anaesthesia. Hair was shaved, and the skin prepared with antiseptic solution. A curved incision was made above the ear then the temporalis muscle was elevated. A craniectomy was performed. The dura was opened and then a corticectomy for access to the lesion with mild temporal lobe retraction. The lesion was initially debulked, and then the densely adherent capsule was removed (Figure 3). Once the intracranial component was removed, the mastoid cavity debris was evacuated. The previous modified radical mastoidectomy was adequate and no revision of this was required. Part of the craniectomy bone piece was completely wrapped in temporalis fascia and used to seal the tegmen defect from above. The fascia allowed a “snug” fit within the tegmen defect, reducing the potential for CSF leak or future problems. The temporal lobe was then allowed to return to its normal position over the temporalis wrapped bone graft. The dura was closed, temporalis muscle was replaced, and the skin was closed in layers. A head bandage was placed for 48 hours.

Histopathology and culture confirmed the lesion as a cholesteatoma infected with* Aeromonas hydrophila*. The patient continued intravenous vancomycin, oral ciprofloxacin, and topical ciprofloxacillin-hydrocortisone ear drops postoperatively. There were no postoperative complications. She was later discharged with a 6-week course of intravenous vancomycin and oral ciprofloxacin. The patient remains well with no complications over 12 months postoperatively.

## 3. Discussion

The incidence of intracranial extension of acquired cholesteatoma is approximately 1.25% with the majority propagating into the middle cranial fossa and a minority to the posterior cranial fossa [7]. The most frequent route of intracranial extension of acquired cholesteatomas is through the supratubal recess or anterior epitympanic air cells [7]. According to Horn's series [7], all patients with middle cranial fossa extension of cholesteatoma via the supratubal recess had horizontal facial nerve involvement whilst none had intradural involvement. In contrast, our patient had intradural extension and no facial nerve involvement. This is similar to another report by Habesoglu et al. [8], where a patient with large intradural cholesteatoma had no facial nerve palsy.

It is obvious that our patient's cholesteatoma spread directly through a bony dehiscence of the tegmen tympani and a defect in the dura. This may have occurred due to the original cholesteatoma or by iatrogenic injury during the prior mastoidectomy, a mechanism hypothesized by Quaranta et al. [9]. The bone eroding capability of cholesteatoma via osteoclast activation is well recognized, and endotoxin release from bacterial biofilms in cholesteatoma may further promote the osteolytic process [10].

Anaerobic bacteria are encountered in 67% of acquired cholesteatoma [6]. This includes* Bacteroides *spp. and the facultative anaerobes such as* Streptococcus *spp. and MRSA.* Pseudomonas aeruginosa *is the most common aerobe [5, 6]. The coinfection of anaerobes and aerobes are observed in 50% of acquired cholesteatomas [6].* A. hydrophila* inhabits aquatic environments in more frequent numbers in warmer climates which corresponds to the patient's residence at Urbenville, New South Wales, a small rural town. The organism is known to reside in rain water tanks which are the only source of water in such rural areas. On further enquiring, she admitted to using water from a rain water tank at home for cooking, for showering, and often to syringe her ears. We suspect her use of this source of water for ear syringing had led to the bacterium gaining access to the mastoid cavity and infecting the cholesteatoma.

Otorrhoea was the main presenting complaint in the majority of the intracranial acquired cholesteatoma patients reported by Horn [7]. Similar to our patient, headaches are common in patients with intradural acquired cholesteatoma [8, 9]. The majority have hearing loss [7, 11]. Facial palsy, tinnitus, vertigo, and imbalance can be observed in patients who have medially propagating cholesteatoma [7, 11]. As with our patient, tympanomastoidectomy or modified radical mastoidectomy 20 or more years prior to presentation has been associated with large intracranial cholesteatomas [8, 12]. This reflects the chronic slow growing nature of these lesions.

CT scans enable identification of the tegmen tympani bony dehiscence and intracranial extension point. MRI is utilised for accurate soft tissue differentiation. Cholesteatoma usually appears hypointense or isointense in T1-weighted MRI and hyperintense on T2-weighted MRI [13] and isointense with gadolinium contrast enhancement on T1-weighted MRI. In our patient's T1-weighted MRI with gadolinium contrast scan, there was a ring enhancing border of lesion. T2-weighted MRI demonstrated cytotoxic oedema of surrounding brain tissue. These factors raised suspicion of secondary superinfection of lesion causing localised cerebral inflammation.

A combined transmastoid/middle fossa approach can be used to remove large acquired cholesteatoma that has extended to the middle fossa. The majority of patients only require one operation to remove the lesion, as evidenced in a 13 patient case reviews of intracranial cholesteatomas by Burggraaff et al. [11]. Most patients who had preoperative normal facial nerve function (House-Brackman I) preserved function postoperatively [11, 14]. The prognosis of petrous cholesteatoma surgery remains positive. In the current largest series of 43 patients by Moffat et al., there have been no perioperative deaths and no long-term recurrence in 95.4% of patients with a median follow-up of 10 years [14]. Perioperative MRSA positive patients are at a higher risk of postoperative otorrhoea [5].

## 4. Conclusion

We have presented a large recurrent, acquired cholesteatoma with intracranial extension to the temporal lobe harbouring* A. hydrophila *causing localised cerebral inflammation. Persistent otorrhoea, fever, and neurological symptoms were experienced by the patient at presentation with a relatively asymptomatic progression of disease for over 20 years. A transmastoid/middle fossa approach enabled successful removal of the cholesteatoma. The patient has made an uncomplicated recovery. In this case, tegmen tympani dehiscence and a presumed dural breach at the time of surgery 23 years priorly allowed such massive intradural extension.
